# Leveraging 5G technology for robotic surgery and cancer care

**DOI:** 10.1002/cnr2.1595

**Published:** 2022-03-09

**Authors:** Krunal Pandav, Austen G. Te, Nir Tomer, Sujit S. Nair, Ashutosh K. Tewari

**Affiliations:** ^1^ Department of Urology Icahn School of Medicine at Mount Sinai New York NY USA; ^2^ Laboratory of Biochemical Genetics and Metabolism The Rockefeller University New York NY USA

**Keywords:** cancer care, robotic surgery, surgical oncology, surgical therapy, urologic oncology, 5G

## Abstract

**Background:**

The field of robotic surgery has seen significant advancements in the past few years and it has been adopted in many large hospitals in the United States and worldwide as a standard for various procedures in recent years. However, the location of many hospitals in urban areas and a lack of surgical expertise in the rural areas could lead to increased travel time and treatment delays for patients in need of robotic surgical management, including cancer patients. The fifth generation (5G) networks have been deployed by various telecom companies in multiple countries worldwide. Our aim is to update the readers about the novel technology and the current scenario of surgical procedures performed using 5G technology. In this article, we also discuss how the technology could aid cancer patients requiring surgical management, the future perspectives, the potential challenges, and the limitations, which would need to overcome prior to widespread real‐life use of the technology for cancer care.

**Recent findings:**

The expansion of 5G technology has enabled some countries to conduct remote surgical procedures, tele‐mentored and real‐time interactive procedures on animal models, cadavers, and humans, demonstrating that 5G networks could offer a potential solution to previously experienced latency and reliability hurdles during the remote surgeries performed in the 2000s.

**Conclusion:**

New technological advancements could serve as a ground for emerging novel therapeutic applications. While limitations and challenges related to the 5G infrastructure, cost, compatibility, and security exist; researching to overcome the limitations and comprehend the potential benefits of integrating the technology into practice would be imminent before widespread clinical use. Remote and tele‐mentored 5G‐powered procedures could offer a new tool in improving the care of patients requiring robotic surgical management such as prostate cancer patients.

## 1 INTRODUCTION

Significant advancements have been made in the field of robotic surgery in the past few years.^1^, ^2^ Robotic surgery is preferred for urologic, gynecologic, thoracic, cardiothoracic, and gastrointestinal procedures.^3^ Moreover, many large hospitals across the United States^4^ and the countries around the world^4^, ^5^ have adopted robotic surgery as a standard. The emergence of robotic surgery has created a significant impact on the surgical management of prostate cancer (PCa)^6^ making robotic assisted radical prostatectomy (RARP) a standard procedure for localized PCa.^2^ The use of robotic surgery the hospitals in Michigan increased 8.4‐fold in 6 years (from 1.8% in 2012 to 15.1% in 2018), demonstrating how robotic surgery continues to diffuse among common surgical procedures.^7^

Remote robot‐assisted surgery was first successfully conducted^8^ in 2001 but has not been integrated into clinical practice due to technological barriers, including high latency. The lack of surgical expertise in rural areas is a problem faced by various countries.^9^, ^10^, ^11^ In addition, the location of robotic surgeons in urban areas may increase the travel burden and cause treatment delay in cancer patients requiring robot‐assisted surgical management.^12^ These shortages could be addressed by tele‐surgery,^13^ which utilizes the real‐time communication and exchange of medical information, including image, audio, and video, to be digitized and transmitted via telecommunication networks, allowing surgeons in an urban location to conduct a real‐time procedure.^14^

Currently, the deployment of fifth‐generation (5G) networks is in progress in multiple countries worldwide. The expansion of the novel technology has enabled clinicians to conduct remote procedures, tele‐mentored surgeries, and real‐time interactive surgeries on animal models, cadavers, and humans. In the following article, the authors discuss detail the history of robotic surgical systems, a summary of the internet and the 5G networks, and the current scenario of remote procedures using the 5G network with an aim to update the readers about the novel technology, illustrate the efficacy and feasibility of the use the novel technology and potential benefits for the cancer patients requiring surgical interventions.

## 2 HISTORY OF SURGICAL ROBOTIC SYSTEMS

Robotic surgical systems have been in development for over 40 years. Arthrobot, developed and used in Vancouver in 1983, was the world's first surgical robot.^15^, ^16^

The PUMA 200 was a robotic system used initially for industrial purposes. In 1985, researchers released a better and improved version of PUMA 200 called the PUMA 560, both made by Unimation Limited.^17^ It was a programmable robot whose computer was compatible with varied imaging computers used in past biomedical fields. Numerous tests were conducted on the robotic system to calibrate and test the accuracy, including tests on a chessboard and watermelons. A neurosurgical procedure that involved holding the fixture close to the patient's head was conducted using PUMA 560. The surgeon used the fixture to guide drills and biopsy probes, making it the first robot‐assisted surgical procedure to conduct a brain tissue sample biopsy under CT guidance. However, PUMA 560 had drawbacks, including high setup times, accuracy, and safety issues.^17^, ^18^, ^19^ In 1988, transurethral resection of the prostate (TURP) was performed using the PUMA 560 and was considered to be the first urologic use of a medical robot.^20^

PROBOT was developed in England and utilized in prostate reconstruction and transurethral prostate resection surgeries.^21^, ^22^ In 1992, the ROBODOC, developed by the Integrated Surgical Supplies, Inc., became the first US Food and Drug Administration (FDA) approved surgical robot. The ROBODOC was developed to perform hip replacements, specifically.^21^, ^23^, ^24^, ^25^

Through the late 1980s and early 1990s, Computer Motion developed their robotic system named AESOP, Automatic Endoscopic System for Optimal Positioning. AESOP assisted surgeons by providing a steady operating field without the risk of a fatigued or inexperienced scope holder. AESOP controlled the orientation of the laparoscope initially using a foot pedal. Later the orientation was controlled using voice commands. AESOP received FDA approval for intra‐abdominal surgeries in 1994 and became the first FDA‐approved robotic device for intra‐abdominal procedures.^21^, ^26^ ZEUS was the second‐generation robotic system introduced by Computer Motion. It provided instrument and camera control, had three robotic arms and a 2D video screen. Out of the three arms, one arm was for a 2D laparoscope and two other arms were to control the surgical instruments. The surgeon used a remote console to control the instrument arms and similar to the AESOP, the camera could be operated with voice commands The surgeon's movements were translated into the laparoscopic instruments with the help of a computer.^8^, ^21^, ^27^ In September 2001, the first transatlantic robot‐assisted telesurgery was conducted successfully using ZEUS, which laid the foundation of remote surgery, demonstrating the feasibility and medical potential to provide surgical support to rural and international areas. It was a robot‐assisted laparoscopic cholecystectomy on a porcine model, transmitting the signals between Strasbourg, France, where the animal models were located, to New York, where the surgeon was located.^8^

While ZEUS gained significant popularity, the da Vinci robotic system, developed by American company Intuitive Surgical, Inc., was shown to have a shorter learning curve than ZEUS and more intuitive technical movements. The da Vinci robotic system was approved by the FDA in 2000 for general laparoscopic procedures.^28^ The system includes a surgeon master console, a four‐armed surgical robot on a patient trolley, an imaging system and is approved for a wide variety of surgical procedures.^29^ Using the da Vinci robotic system, surgeons in the United States conducted four right laparoscopic transcontinental telesurgical nephrectomies in porcine models in 2008.^30^ Animal subjects were located in Sunnyvale, California, and the surgeon was located in two different locations: Cincinnati, Ohio (approximately 2400 miles from Sunnyvale) & Denver, Colorado (approximately 1300 miles from Sunnyvale). The da Vinci surgical system was utilized in all four of these surgeries and performed via a wired internet connection to study the efficacy and reliability of remote surgery. Cincinnati Bell provided internet connection, and commercial video code/decoding protocols were provided by Polycom and Haivision for remote control, visualization, and audio. The wired connection transferred data at 3 and 8 Mbps connection for Cincinnati/Sunnyvale and Denver/Sunnyvale. The Cincinnati connection had an average latency of 900 ms with poor visualization, while the Denver connection had an average latency of 450 ms with adequate visualization.^30^

The robotic‐assisted surgical device space is growing rapidly with the development and expansion of various devices, including Ion (Intuitive Surgical), Mako (Stryker), NAVIO (Smith & Nephew), and Monarch (Auris Health)^31^ and many others which have been utilized in real‐time remote procedures discussed in the following sections.

## 3 BACKGROUND OF THE INTERNET AND 5G

**FIGURE 1:**
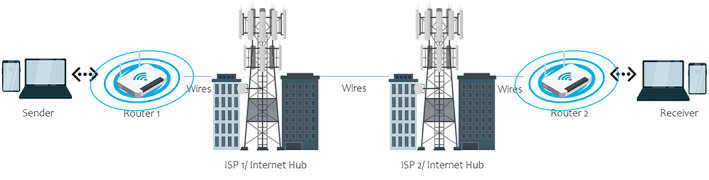
Simplified visual of representation of how devices transfer data via wired/fixed‐broadband connections using Wi‐Fi routers. The sender sends the message in a binary language signal to the router. The router will transfer the signal to the internet service provider's (ISP) station and internet hub via cables. The signal travels to the receiver's ISP and internet hub. From there, the signal travels to the receiver's device via cables to the router

**FIGURE 2:**
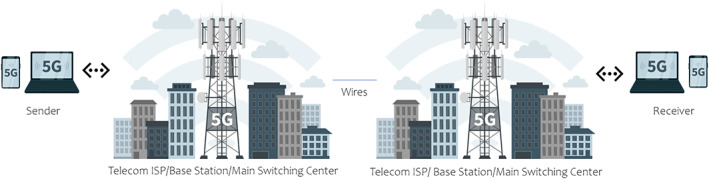
Simplified visual of representation of how devices transfer data via wireless 5G mobile connections. The sender sends the message via smartphone in a binary language to an internet service provider (ISP) tower wirelessly through radio frequency (RF) electromagnetic waves. The ISP tower is connected to a base station that transfers the signal through cables to the receiver's base station. The signal reaches the receiver's device via RF waves

A simplified visual representation of how devices transfer data via wired or broadband (Figure 1) and wireless (Figure 2) connections is shown in the figures. Advancements in 2G, 3G, and 4G mobile networks have drastically improved wireless internet services.^32^ 5G networks provide a high data transfer rate at 10 GB/s.^33^ 4G networks utilize a system that incorporates every aspect into one network and functions as one body.^33^, ^34^ On the other hand, 5G networks have developed a “network splicing/slicing” scheme that divides the network architecture into multiple networks specialized in one specific function.^33^, ^34^ Splicing allows the 5G network to optimize resources toward the relevant functions being used. These advancements allow 5G networks to achieve higher data transfer speed, communication, reliability, and ultra‐low‐latency than 4G networks.^32^, ^33^, ^34^

It is essential to understand latency. Latency is the response delay between a device and the hosting server or target device, which is affected by the data transfer rate of a network and the amount of data needed to be transferred. In remote surgery, these characteristics are essential for the surgeon to respond adequately to changing circumstances and the quality of the video stream to provide appropriate information. Inadequate time or poor video quality could lead to surgical trauma and complications for the patient. The maximum resolution possible for Dr. Marescaux and colleagues, in their surgery conducted in early 2000s, was 1024 pixels by 768 pixels and a data transfer rate of 10 Mbps.^8^ A maximum latency for successful surgery at 330 ms was established but conducted most of the surgery at an average latency of 155 ms.^8^ Better video quality and faster data transfer speed would be needed to improve the success of remote surgery. The increasing resolution will increase the amount of data required to be transferred but improve the video quality for the surgeon to conduct the surgery accurately.

Similarly, data transfer speed increases can help the surgeon reduce the latency and allow the surgeon to respond more quickly. The DaVinci robot is capable of at least 1920‐pixel by 1080‐pixel resolution (about 2.6 times larger than Dr. Marescaux), and 5G networks can reduce both the latency and improve video stream quality through their maximum transfer rate of 10Gb/s. Concerning surgeons, Zheng and colleagues found the average endoscopic surgeon's response time to their most complex scenario was 397 ms ± 19 s.^35^ Most successful remote surgeons utilized average latencies between 76 and 150 ms for a potential approximate response time of 475–660 ms.^36^, ^37^, ^38^ As such, we can speculate the maximum safe latency period to be around 150 ms, which is approximately a 37.5% increase in normal surgeon response time. Thus, to data transfer speeds from improved mobile networks can lead to lower latencies or improvements in resolution.

## 4 CURRENT SCENARIO OF THE USE OF 5G IN ROBOTIC SURGERY

Many countries are slowly adopting 5G networks.^39^ In addition, the expansion of surgical robot technologies and 5G network systems has enabled some countries to conduct remote robotic procedures using 5G. In the following paragraphs, we present different robotic procedures, which involve the use of 5G technology.

**FIGURE 3:**
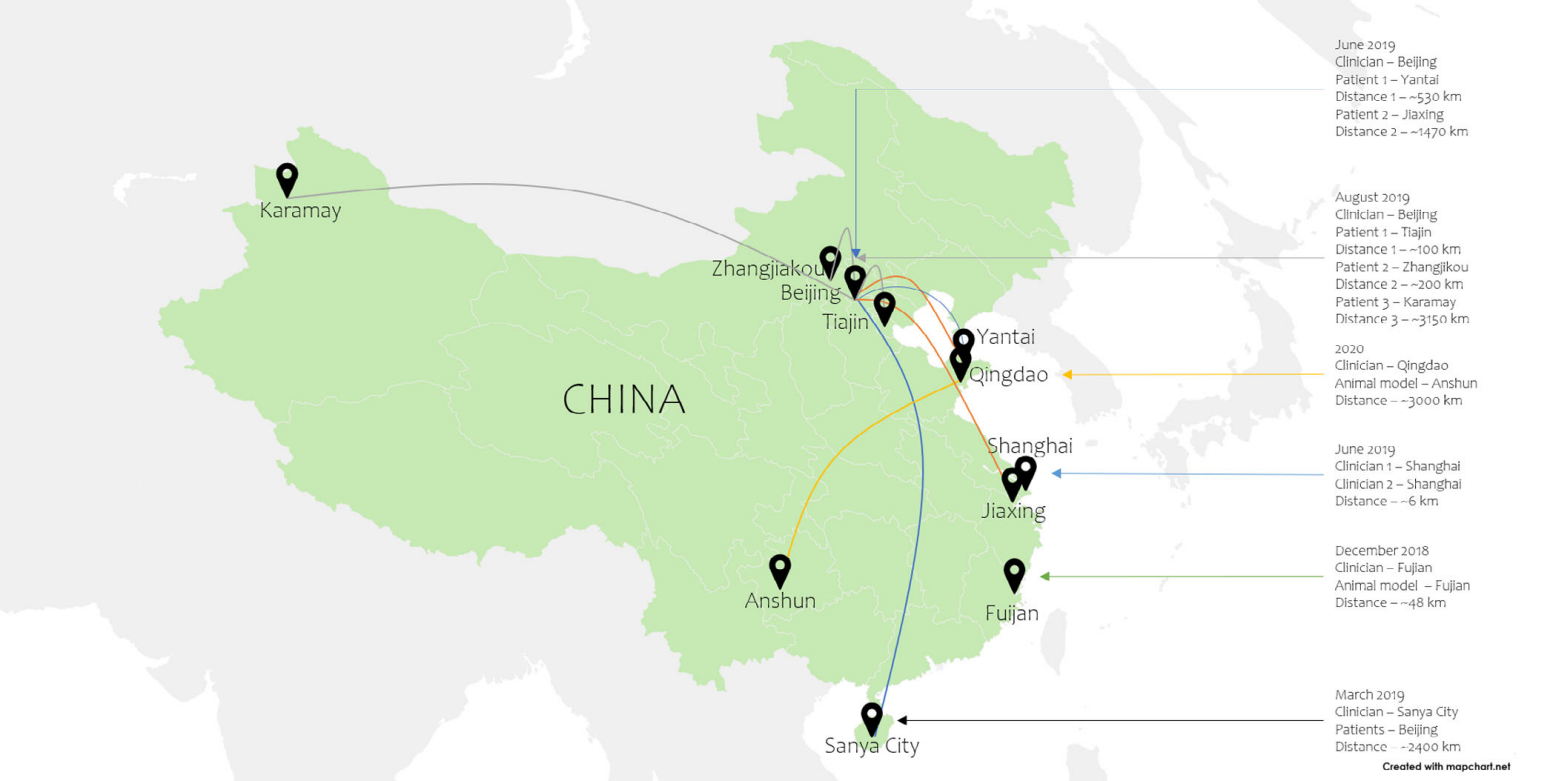
Procedures performed using 5G in PRC

In December 2018, a remote hepatectomy in a porcine model was performed at the Fujian, People's Republic of China (PRC) using the Kangduo robotic surgery system and 5G network by Huawei Technologies Company and China Unicom. The equipment control was located in the Fujian Branch of China Unicom, and the operating theater was located in Mengchao Hepatobiliary Hospital in Fujian, Fuzhou, PRC, approximately 48 km (30 miles) away. Two robotic arms (bipolar coagulation and electrocoagulation hooks) and lens were controlled remotely by the surgeon to resect a 2 cm × 2 cm × 3 cm portion of the liver. The surgery lasted for around 60 min, with a total blood loss of approximately 5 ml. In addition, average latency was reported at less than 150 ms^40^ (Figure 3).

**FIGURE 4:**
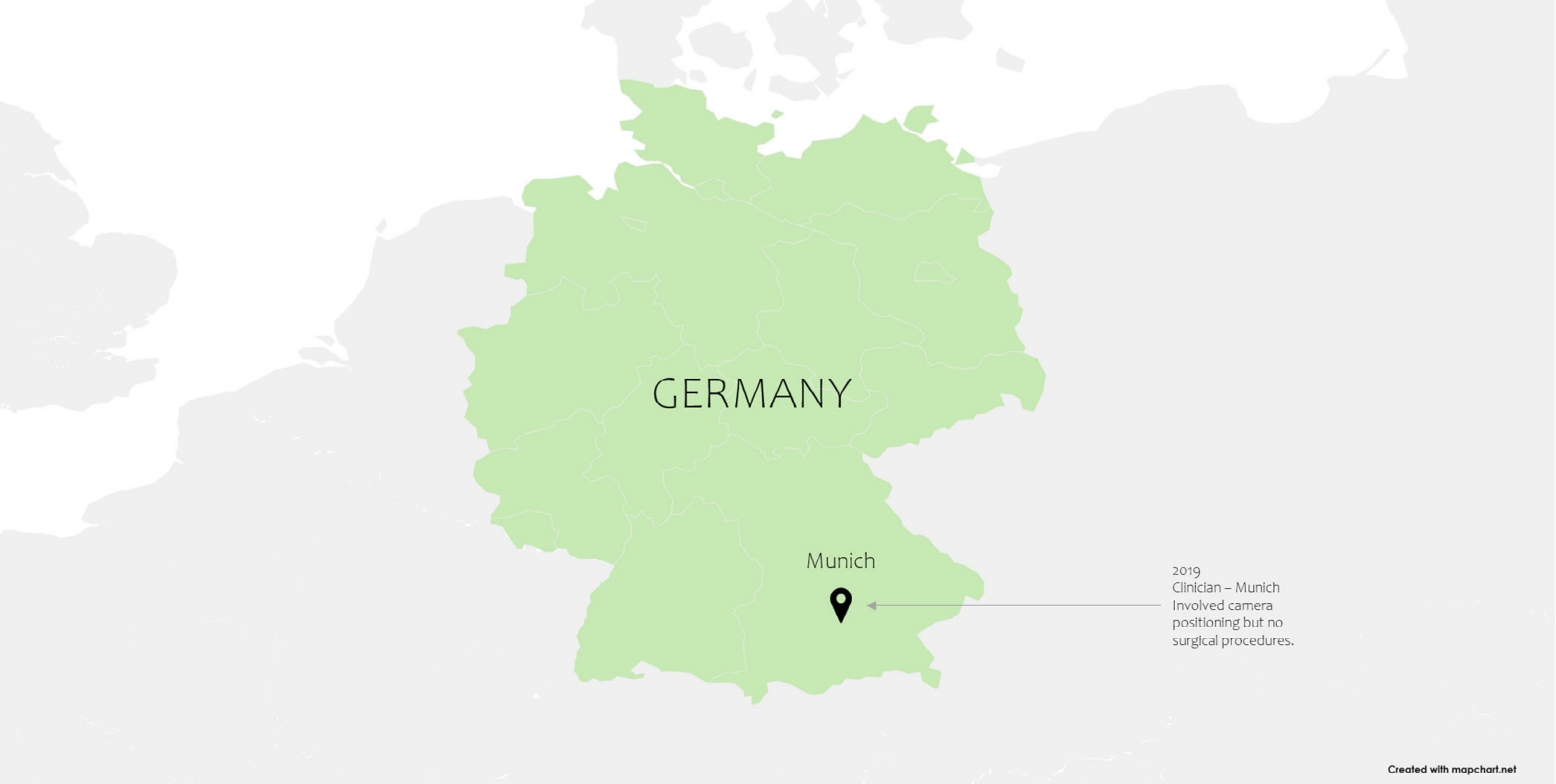
Procedure performed using 5G in Germany

In 2019, a study to evaluate the performance of 5G in two different medical applications was conducted in Munich, Germany, and involved camera positioning. The video streaming rate was 900 KB–1 Mbps (7.2–8 Mbps), and the robotic control command rate was 2.4–7.2 KB/s (19.2–57.6 KB/s). The latency was 2–60 ms, and it depended on the transmitted data packet length. A Delphi study was also conducted, which showed that the participants agreed that 5G has a great potential in the healthcare domain and needs to be further explored^41^ (Figure 4).

In March 2019, clinicians remotely controlled implants for deep brain stimulation (DBS) through 5G to treat Parkinson's disease and brain ailments. Three patients (two Parkinson's disease, one essential tremor) were recruited and operated on‐site to install the headframe, craniotomy, and microelectrode puncture in Beijing. The remote‐control group controlled the microelectrode recording and imaging to confirm the implantation of the electrode. The remote‐control group was located approximately 2400 km away (Hainan Hospital of Chinese PLA General Hospital, Sanya City, PRC) from the operating site (Beijing, PRC). The 5G network utilized in this study was through Huawei and China Mobile. During the procedures, peak download speed was reported at 119 Mbps with an average latency of 76 ms. At the 3‐month follow‐up, it was recorded that the patients improved by 43.6%, 84.9%, and 90.5%, respectively, according to the Unified Parkinson's Disease Rating Scale (MDS)^42^ (Figure 3).

**FIGURE 5:**
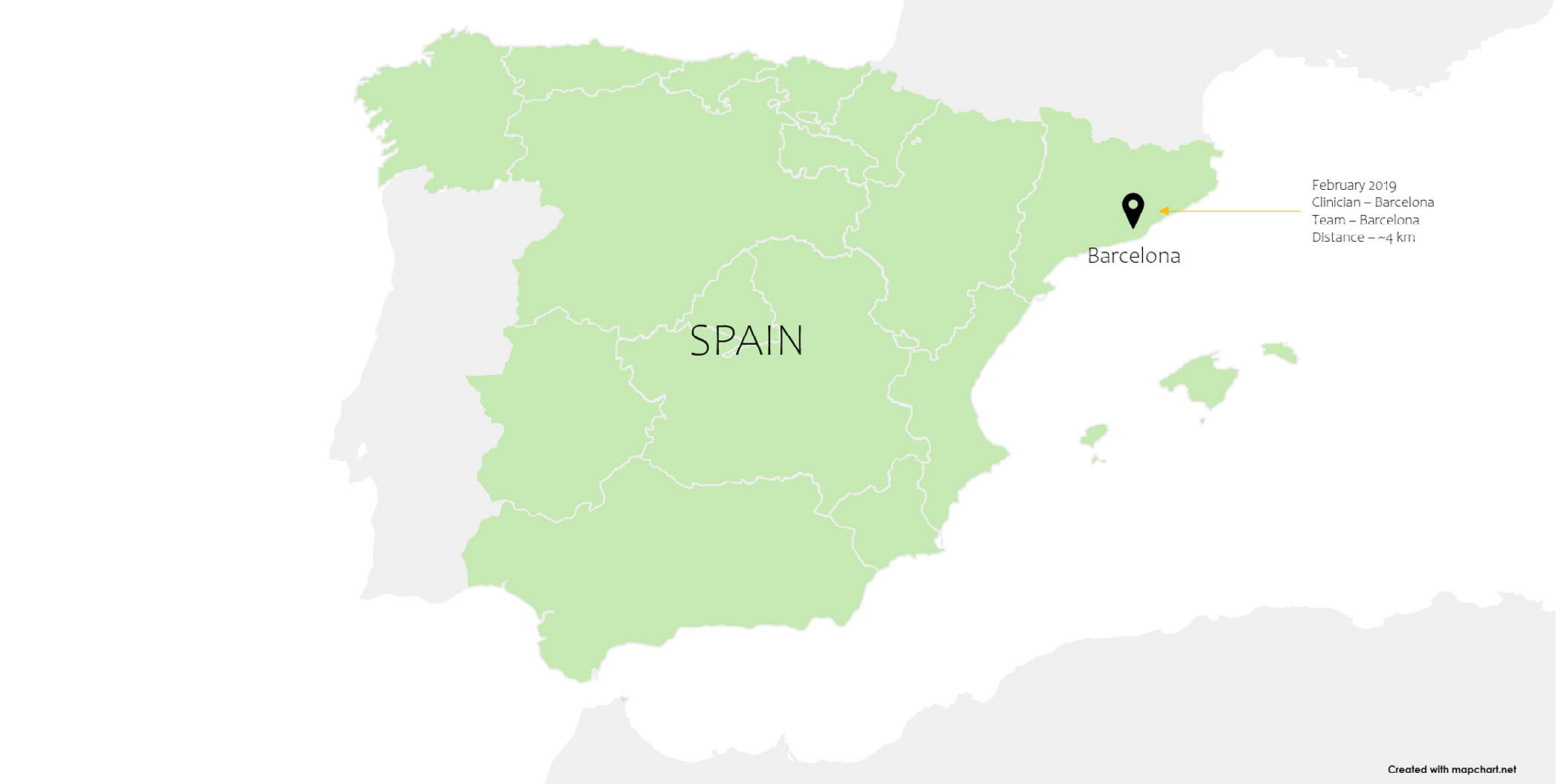
Procedures performed using 5G in Spain

In February 2019, the first tele‐mentored surgery over 5G^43^ was done in Barcelona, Spain.^44^, ^45^, ^46^ The laparoscopic access with a medial‐to‐lateral approach was performed between the Mobile World Congress (MWC) Barcelona 2019 at Fira Gran Via and the Hospital Clinic de Barcelona, approximately 4 km away. The telementored surgery was conducted with the help of a 5G connection provided by Vodafone and an online education portal, the Advances in Surgery (AIS) Channel. The procedure duration was around 118 min, the average latency time was 202 ms, and the mean data upload speed was 98 Mbps, with no observable signal loss, image buffering, or pixelation. Three surgeons rated the image quality as 9.67/10 and audio exchange as a 10 out of 10^44^, ^45^, ^46^ (Figure 5).

In June 2019, remote tele‐assisted surgery was done between MWC Shanghai 2019, Shanghai New International Expo Center (SNIEC) in Shanghai, PRC, and Shanghai East Hospital, approximately 6 km away. Surgeon 1 provided live guidance to Surgeon 2 via the 5G connection provided by China Mobile. The surgery lasted around 138 min, with an average latency of 148 ms. The mean data upload speed was 101 Mbps with no observable signal loss. Image quality was graded as a 9.5/10, and the audio exchange was again 10 out of 10^36^, ^44^, ^47^ (Figure 3).

In June and August 2019, two surgeries with a total of 12 orthopedic telerobotic spinal placement on patients (four thoracolumbar fractures, six lumbar spondylolistheses, and two lumbar stenoses) were conducted. The first procedure, the “one‐to‐two” simultaneous remote orthopedic pedicle screw placement, was conducted in June 2020 between Beijing Jishuitan Hospital, Shandong Yantaishan Hospital, and Zhejiang Jiaxing Second Hospital.^37^ The second procedure, the “one‐to‐three” simultaneous remote orthopedic pedicle screw placement, was conducted in August 2020 between Beijing Jishuitan Hospital, Tianjin First Central Hospital, Second Hospital of Zhangjiakou City, and Karamay Central Hospital.^37^ In both surgeries, the master control room and surgeon responsible for the operations were located in Beijing Jishuitan Hospital, while the other hospitals were the surgery location. The 5G network utilized was provided and established by China Telecom and Huawei Technologies, and the surgical robot used was the TiRobot system. The distance between the master room and the various operating theaters was 114 to 3154 km.^37^ The average surgery time was 142.5 ± 46.7 min; guide pin insertion time was 41.3 ± 9.8 min. A total of 62 screws were implanted, and the average difference in screw placement was 0.76 ± 0.49 mm. No intraoperative adverse events after surgery were observed. However, there was one complication where a lumbar spondylolisthesis patient had a CSF leak a day after surgery and it was considered to be related to a nerve decompression procedure^37^ (Figure 3).

**FIGURE 6:**
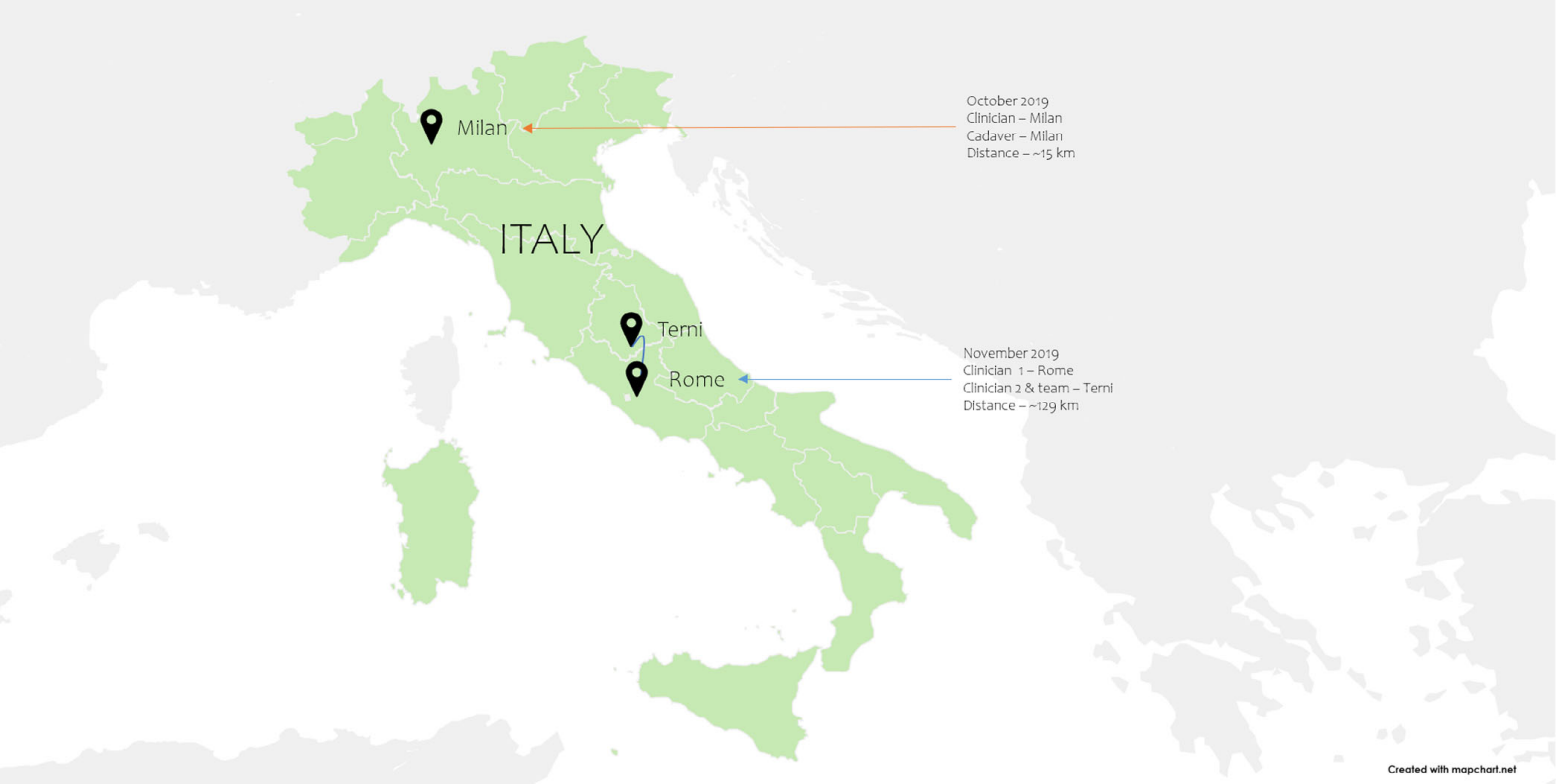
Procedures performed using 5G in Italy

In October 2019, transoral laser microsurgery procedures (ventriculotomy, type 1 cordectomy, and type 4 cordectomy) were conducted on an adult human cadaver. The otolaryngologist was located at Vodafone Village, Milan, Italy, while the cadaver was in the San Raffaele Hospital, Milan, Italy, approximately 15 km away. The computer‐assisted laser microsurgery system (CALM) was used to control a medical CO_2_ laser (SmartXid2 C60 DEKA), Panda robot (robotic surgical forceps by Frank Emika), and a VITOM 3D stereo exoscope (Karl Storz). Support staff set up the cadaver and managed the laser settings. The 5G network utilized in this study was the 5G Radio Access Network with a 1 Gbps bandwidth by Vodafone. Throughout the lecture, the average latency observed for 3D high‐definition video was 102 ± 9 ms, with a maximum latency of 280 ms and a mean round trip delay between devices estimated at 40 ms. The surgical evaluation was deemed highly precise after procedures were conducted^38^ (Figure 6).

In November 2019, a live interaction surgery using 5G was performed from a distant location. Surgeon 1, who was located in Rome, Italy, was able to view laparoscopic gastrectomy in Santa Maria Hospital in Terni, Italy performed by Surgeon 2 and his team via the visual‐reality visor. The patients' biometrics via visor connected to three cameras, including one super high‐definition 360° camera, in the operating room. In addition, the laparoscopic camera allowed viewing organs. The event was presented live at the 30th International Congress of Digestive Surgery and was watched by over surgeons present at the Congress and over 30 000 surgeons worldwide via online video streaming platforms^48^, ^49^ (Figure 6).

Furthermore, four laparoscopic procedures: left nephrectomy, partial hepatectomy, cholecystectomy, and cystectomy, were performed on 25 kg porcine models in 2020. Surgeons were located in Qingdao, PRC, and porcine models were in Anshun, PRC, approximately 3000 km away.^50^ The “MicroHand” surgical robot was used, assisted by the Hisense computer‐assisted system (CAS) for remote access. They compared a wired 100 Mbps wired China Unicom connection to a 5G 1Gbps China Unicom connection during the procedure.^50^ Average latency of 264 ms with a round trip delay of 114 ms and a 1.2% packet loss ratio was noted by the 5G network. The “control” wired connection had an average latency of 206 ms with a round trip delay of 56 ms. The total duration was 120 min, and the total blood loss was 25 ml^50^ (Figure 3). The details of the procedures discussed above are summarized in Table 1.

**TABLE 1:** Procedures performed using 5G

Reference	Month/year of procedure(s)	Summary of the procedure(s)	Procedure duration	Location of clinician(s)	Location of clinician's team/2nd clinician(s)/patient(s)/animal model/cadaver	Distance^a^	Latency	5G telecom service provider(s)	Robotic surgery device
[40]	December 2018	Hepatectomy on a porcine model (bleeing ~5 ml)	60 min	Clinician location: Fujian Branch of China Unicom, Fujian, Fuzhou, PRC	Animal model location: Mengchao Hepatobiliary Hospital, Fujian Medical University, Fujian, Fuzhou, China	~48 km (~30 miles)	<150 ms	1. Huawei Technologies Company 2. China Unicom	Kangduo Robotic Surgery System (Kangduo Robot Co., Ltd.)
[41]	2019	Evaluation of the performance of 5G	NP	Clinician location: Munich, Germany	Involved camera positioning but no surgical procedures	NP	2–60 ms	Local setup that is, p2p using 5G transmission technology, provided by Huawei	SoloAssist (AKTORmed)
		Person/asset track & tracing							
		Video data transmission for telesurgery							
		Delphi study							

[6, 43, 44, 45, 46, 47]	February 2019	First 5G tele‐mentored surgery—endoscopic local excision of a rectal polyp on a 65‐year‐old male patient	118 min	Clinician location: MWC Barcelona 2019, Fira Barcelona Gran Via, Barcelona, Spain	Team location: Hospital Clinic de Barcelona, Barcelona, Spain	Approximately 4 km (2.48 miles)	Average latency time of 202 ms	1. Vodafone 2. Advances in Surgery (AIS) Channel (an online education portal)	NP
	June 2019	First 5G tele‐assisted surgery in Asia—a 50‐year‐old female undergoing a laparoscopic low anterior resection with natural orifice specimen extraction	138 min	Clinician 1 location: MWC Shanghai 2019, SNIEC, Shanghai, PRC	Clinician 2 location: Shanghai East Hospital, Shanghai, PRC	Approximately 6 km (3.72 miles)	Average latency of 148 ms	China Mobile	NP
[42]	March 2019	Deep brain simulation device placement. Three patients (two Parkinson's disease, one essential tremor) were recruited and operated on‐site to install the headframe, craniotomy, and microelectrode puncture in Beijing. The remote‐control group controlled the microelectrode recording and imaging to confirm the implantation of the electrode	NP	Clinician location: Hainan Hospital of Chinese PLA General Hospital, Sanya City, Hainan, PRC	Patients' location: Beijing, PRC	Approximately 2400 km (1491 miles)	Average latency—76 ms	1. Huawei Technologies 2. China Mobile	NP
[37]	June 2019	“One‐to‐two” simultaneous remote orthopedic robot‐assisted procedure (pedicle screw placement)	142.5 ± 46.7 min	Clinician location: Beijing Jishuitan Hospital, Beijing, PRC	Patient location: 1. Shandong Yantaishan Hospital, Yantai, Shandong, PRC 2. Zhejiang Jiaxing Second Hospital, Jiaxing, Zhejiang, PRC	1. Approximately 530 km (329.3 miles) 2. Approximately 1470 km (913.4 miles) (114–3154 km)	28 ms	1. China Telecom (Beijing, China) 2. Huawei Technologies Co., Ltd. (Shenzhen, China)	TiRobot system (co‐designed by Beijing Jishuitan Hospital and TINAVI Medical Technologies Co., Ltd.)
	August 2019	“One‐to‐three” simultaneous remote orthopedic robot‐assisted procedure (pedicle screw placement)	142.5 ± 46.7 min	Clinician location: Beijing Jishuitan Hospital, Beijing, PRC	Patient location: 1. Tianjin First Central Hospital, Tianjin, PRC 2. Hebei Zhangjiakou Second Hospital, Hebei, PRC 3. Xinjiang Karamay Central Hospital, Karamay, Xinjiang, PRC	1. Approximately 100 km (62.1 miles) 2. Approximately 200 km (124.2 miles) 3. Approximately 3150 km (1957.3 miles) (114– 3154 km)	28 ms	1. China Telecom (Beijing, China) 2. Huawei Technologies Co., Ltd. (Shenzhen, China)	TiRobot system (co‐designed by Beijing Jishuitan Hospital and TINAVI Medical Technologies Co., Ltd.)
[38]	October 2019	Transoral laser microsurgery procedures on a human cadaver 1. Ventriculotomy 2. Type 1 cordectomy 3. Type 4 cordectomy	NP	Clinician location: Vodafone Village, Milan, Italy	Cadaver location: San Raffaele Hospital, Milan, Italy	Approximately 15 km (9.3 miles)	102 ± 9 ms (maximum latency 280 ms)	Vodafone Italia (5G Radio Access Network with a 1 Gbps bandwidth)	1. Computer‐assisted laser microsurgery system (CALM)—to control a medical CO_2_ laser (SmartXid2 C60 DEKA) 2. Panda robot (robotic surgical forceps by Frank Emika) 3. VITOM 3D stereo exoscope (Karl Storz)
[48, 49]	November 2019	The first worldwide immersive surgery (laparoscopic gastrectomy) on a human patient	NP	Clinician 1 location: Auditorium del Massimo in Rome, Italy	Clinician 2 and team location: Saint Mary's Hospital in Terni, Italy	Approximately 129 km (80.1 miles)	NP	TIM 5G	NP
[50]	2020	Surgeries on porcine models of around 25 kg 1. Left nephrectomy 2. Partial hepatectomy 3. Cholecystectomy 4. Cystectomy	120 min	Clinician location: Qingdao, Shangdong, PRC	Animal model location: Anshun, Guizhou, PRC	Approximately 3000 km (1864.1 miles)	Average latency of 264 ms for 5G connection	Public 5G wireless network monitored by China Unicom operators in real time	1. “MicroHand” surgical robot system (Shandong Weigao Co., Ltd) 2. Assisted by the Hisense computer‐assisted system (CAS) for remote access

Abbreviations: MWC, Mobile World Congress; NP, not provided; SNIEC, Shanghai New International Expo Center; PRC, People's Republic of China

^a^ Exact distance may vary.

Stable 5G networks have made robotic surgeries and telementoring via 5G networks a reality in some countries. Surgeons in PRC, Germany, Spain, and Italy have demonstrated that robotic procedures using 5G can safely and effectively be conducted. The time to complete the surgeries could be similar to non‐5G robotic surgeries.^37^, ^42^, ^50^, ^51^ These 5G‐powered robotic surgery cases have also demonstrated various backup measures for unstable connections, including partial control by an onsite surgeon and a surgical support staff during the operations. European clinicians have supported that 5G networks can help conduct robotic telesurgery, quantified the image and audio quality of 5G procedures, and reconfirmed the maximum latency threshold at 300 ms.^38^, ^44^ These studies demonstrate against Ullah and colleagues that a highly reliable connection with ultra‐low latency of less than 10 ms is required for robotic surgery.^52^

## 5 POSSIBLE CHALLENGES AND LIMITATIONS

The challenges created by the COVID‐19 pandemic have increased the importance and need for telemedicine. Integrating 5G into healthcare could provide various benefits, including virtual check‐ins, mobile waiting rooms, and encrypted high‐definition medical imagery transfers; creating 5G enabled virtual reality scenarios to reduce pain and anxiety for terminally ill, immunocompromised, or elderly patients.^52^, ^53^, ^54^ However, this integration relies heavily on existing telecom companies to provide widespread coverage. Various telecom companies have already initiated the expansion of 5G coverage in the rural areas in the US.^55^ For example, in December 2020, OpenSignal reported that T‐Mobile 5G users in the rural locations were receiving download speeds of upto 53.4 Mbps.^56^ The speeds and coverage are likely to improve in the coming years all across the country due to the expansion of the network by multiple telecom companies. For instance, in a report published by T‐Mobile in the Forbes, they discuss about covering 90% of rural Americans in the next 6 years.^56^ In addition to a widespread 5G connectivity, 5G‐compatible laptops, cell phones, and robotic surgical systems would be needed.^57^ For 5G robotic surgeries, we must also be aware that a backup surgery team may be required to be onsite if a connection is hindered or lost. In the following surgical cases, we will review their usage of 5G networks and how they managed the limitations of 5G‐powered robotic surgeries.

It is also essential to conduct a cost–benefit analysis before any potential implementation of 5G‐powered robotic surgery for rural areas needing surgeons. From 1981 to 2005, the number of surgeons per 100 000 in rural counties has declined from 7.7 to 5.7 per 100 000^58^ compared to the US average of 55 per 100 000.^59^ The rural number has likely declined further when factoring in hundreds of rural hospitals that have closed and had a 7%–14% reduction in obstetrics from 2009–2017.^60^ In addition, there has been minimal data collected that focuses purely on the financial cost to patients in the US. Canadian studies found that robotic surgery increased procedure costs by about $2700 but failed to factor in the decreased hospitalization stay.^61^ Further studies would help determine the economic feasibility in the concept for both institutions and patients. In addition to the economic feasibility and cost analysis, due to the lack of wide spread use of remote surgery, the opportunity remains for better understanding and assessing the training and learning curve.

## 6 FUTURE PERSPECTIVES

The procedures discussed demonstrate that the latency and reliability difficulties experienced by Marescaux *et al*.^8^ and Sterbis *et al*.^30^ in their remote surgeries in the 2000s, could be overcome by the 5G networks. In addition, after widespread incorporation of the new technology, levering the new technology could fill the need for surgical expertise in rural locations, as surgeons may be able to perform remote robot‐assisted procedures. Studies have shown that some cancer patients, including PCa^12^ and bladder cancer^16^ patients requiring robotic surgical procedures, experience a treatment delay due to increased patient travel distance. The odds of traveling medium to long rage are 1.1–1.2 times higher odds in PCa patients requiring RARP compared to open.^12^ Moreover, there is also an increased need for surgical expertise in rural locations in various countries worldwide.^9^, ^10^, ^11^ Leveraging the new technology could help fill the current lack of surgical expertise and travel time for cancer patients requiring robot‐assisted surgical management.

In the early 2000s, with the development of improved robotic surgical devices, the prospective trial comparing the results of RARP and open retropubic radical prostatectomy by Menon *et al*.,^62^ improvements in the RARP procedure^63^, ^64^ and further development of the Vattikuti Institute prostatectomy^65^, ^66^ paved the way to revolutionizing the surgical management of PCa and incorporation of the novel technology into patient care. In the same way, undoubtedly, there will be further developments in the robotic surgery field and robotic tele‐surgery in the coming years, which could significantly improve patient care. We could expect real‐time virtual reality‐based surgeries and a real‐time three‐dimensional model of cancer lesions and organs during surgeries. With the high‐speed data transfer, artificial intelligence incorporated robotic surgical systems could be also be expected.

In the initial year of implementation, the performance of 5G networks would vary depending on the user's location because 5G uses various frequency bands to operate, namely: high band 5G (transmitting on millimeter‐wave/24–39 GHz frequency), mid‐band 5G (transmitting on 2.5/3.5 GHz frequency), and low band 5G (transmitting on 600–700 MHz frequency).^32^, ^67^ High band 5G connects devices to the closest telecom antenna/base station and can transfer data at the fastest speeds but cover a shorter range and have a poor ability to penetrate obstacles or buildings. On the other hand, mid‐band and low‐band 5G can extend the coverage at the cost of performance. Telecom companies would need to ensure high band wave performance for remote and live‐interactive surgeries, requiring them to install base stations and antennas in close proximity to each other.

## 7 CONCLUSION

Remote tele‐mentored collaboration could help expand knowledge between clinicians in real‐time, increasing medical aid where 5G networks have been established. The high‐speed data transfer could allow large volumes of research and data collection to be shared in real‐time. New technological advancements could serve as a ground for emerging novel therapeutic applications. While limitations and challenges related to the 5G infrastructure, learning curve, cost analysis and device compatibility exist, conducting further research to overcome these limitations and comprehend the potential benefits of integrating the technology before widespread use for cancer management is imminent. Remote and tele‐mentored 5G‐powered procedures could offer a new tool in improving care of patients requiring robotic surgical management such as PCa patients.

## CONFLICT OF INTEREST

Ashutosh K. Tewari has served as a site‐PI on pharma/industry‐sponsored clinical trials from Kite Pharma, Lumicell Inc, Dendreon, and Oncovir Inc. Ashutosh K. Tewari has received research funding (grants) to his institution from the United States Department of Defense, National Institutes of Health, Axogen, Intuitive Surgical, the Arthur M. Blank Family Foundation, and other philanthropy. Ashutosh K. Tewari has served as an unpaid consultant to Roivant Biosciences and advisor to Promaxo. Ashutosh K. Tewari owns equity in Promaxo. Krunal Pandav, Austen G. Te, Nir Tomer, Sujit S. Nair declare no conflicts.

## ETHICAL STATEMENT

Not applicable.

## AUTHOR CONTRIBUTIONS

**Krunal Pandav:** Conceptualization (equal); data curation (lead); formal analysis (lead); investigation (lead); methodology (lead); validation (equal); visualization (equal); writing‐original draft (lead); writing‐review & editing (lead). **Austen G Te:** Data curation (equal); formal analysis (equal); investigation (equal); validation (equal); visualization (equal); writing‐original draft (equal); writing‐review & editing (equal). **Nir Tomer:** writing‐original draft (supporting); writing‐review & editing (supporting); **Sujit S Nair:** Conceptualization (equal); supervision (equal); visualization (equal); writing‐review & editing (equal). **Ashutosh Tewari:** Conceptualization (equal); funding acquisition (lead); project administration (lead); supervision (equal); writing‐review & editing (equal).

## Data Availability

Data sharing not applicable to this article as no datasets were generated or analyzed during the current study.
